# Small airway dysfunction in Chinese patients with idiopathic pulmonary fibrosis

**DOI:** 10.1186/s12890-022-02089-6

**Published:** 2022-08-02

**Authors:** Xinran Zhang, Bingbing Xie, Chenjun Ban, Yanhong Ren, Qiao Ye, Min Zhu, Yan Liu, Shu Zhang, Jing Geng, Dingyuan Jiang, Huaping Dai

**Affiliations:** 1 Department of Clinical Research and Data Management, Center of Respiratory Medicine, China-Japan Friendship Hospital, National Center for Respiratory Medicine; Institute of Respiratory Medicine, Chinese Academy of Medical Sciences, National Clinical Research Center for Respiratory Diseases, Beijing, 100029 China; 2 Department of Pulmonary and Critical Care Medicine, Center of Respiratory Medicine, China-Japan Friendship Hospital, National Center for Respiratory Medicine; National Clinical Research Center for Respiratory Diseases, Institute of Respiratory Medicine, Chinese Academy of Medical Science, Peking Union Medical College, Beijing, 100029 China; 3 Department of Respiration, Dongzhimen Hospital, Beijing University of Chinese Medicine, Beijing, 100027 China; 4 Department of Pulmonary and Critical Care Medicine, Beijing Chao-Yang Hospital, Capital Medical University, Beijing, 100020 China; 5 Department of Respiratory and Critical Care Medicine, China-Japan Friendship Hospital, 2 Yinghuayuan E St, Chaoyang District, Beijing, 100029 China

**Keywords:** Small airway dysfunction, Idiopathic pulmonary fibrosis, Clinical feature, Predictor, Prognosis

## Abstract

**Background:**

Recent years, idiopathic pulmonary fibrosis (IPF) is thought to be a disease of alveoli as well as small airways. This study aimed to demonstrate the clinical feature, predictor, and prognosis of small airway dysfunction (SAD) in Chinese patients with IPF.

**Methods:**

We enrolled 416 patients with IPF who hospitalized in Beijing Chao-Yang Hospital from 2000 to 2014 in this study, and the follow-up ended at December 2016. We collected demographic information, clinical examination results, spirometry results, HRCT results, and blood gas results during the study. Logistic regression analysis was used to identify the predictor for SAD. The COX proportional hazard model was used to analysis the prognosis effect of SAD.

**Results:**

Among all the participants, 165 (39.66%) patients had SAD. FEV1 (% predicted) and FEV3/FVC were significantly associated with SAD in patients with IPF. IPF patients with lower FEV1 (% predicted, OR 30.04, 95% CI 9.61–93.90) and FEV3/FVC (OR 77.76, 95% CI 15.44–391.63) had increased risk for SAD. Patients with SAD were associated with significantly increased risk of mortality in patients with IPF (HR 1.73, 95% CI 1.02–2.92), as well as in IPF patients without other pulmonary comorbidities (COPD, emphysema, and asthma).

**Conclusions:**

Spirometry-defined SAD was like 40% in patients with IPF. Lower FEV1 (% predicted) and FEV3/FVC were main predictors for SAD. IPF patients with SAD showed poorer prognosis.

**Supplementary Information:**

The online version contains supplementary material available at 10.1186/s12890-022-02089-6.

## Background

Idiopathic pulmonary fibrosis (IPF), which is the most common form of the idiopathic interstitial pneumonias, is a progressive, irreversible and fetal lung disease. The main clinical features of IPF are chronic, progressive exertional dyspnea and cough with the histological pattern being usual interstitial pneumonia (UIP). The cause of IPF was unknown, however, the prevalence is increasing annually. Although the natural history of IPF was heterogeneous, the overall survival of patients with IPF is poor [1, 2].

Recent years, small airway dysfunction (SAD) has attracted increasing attention from researchers. The small airways are referred to those with a luminal diameter less than 2 mm [3]. A national cross-sectional study conducted in China estimated that 426 million adults had SAD in 2015 [4]. SAD is thought to precede both the spirometry evidence of COPD and detection of emphysema by CT [5]. In asthma, SAD correlates with the frequency and severity of dyspnea and asthma exacerbations, and might precede the development of asthma [6].

As early as 1977, researchers identified SAD in the lungs of patients with IPF, and provided the histopathological evidence [7]. Subsequent studies have explored the relationship between SAD and IPF [8–10]. Abnormal pathologic, physiological and imaging changes of small airways were also found in Chinese patients with IPF [10]. However, the clinical feature and predictor of SAD in Chinese patients with IPF were still not clear, as well as the prognosis effect of SAD.

To fill that gap, we conducted this study. We aimed to reveal the clinical feature of IPF patients with SAD, to explore the predictor for SAD, and to illustrate the effect of SAD on prognosis of patients with IPF.

## Methods

### Study population

**Fig. 1:**
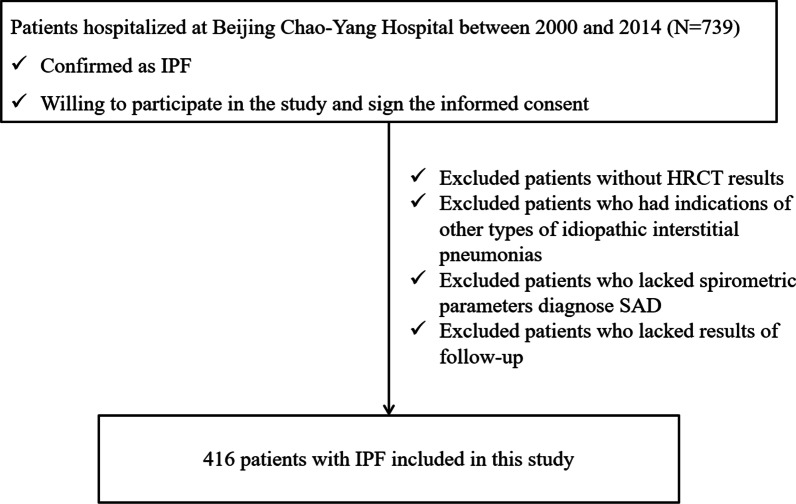
Flow chart of study population selection

Patients with IPF who hospitalized at Beijing Chao-Yang Hospital between 2000 and 2014 were consecutively enrolled in this study. All patients undergo a standard investigation protocol. Finally, 416 patients with IPF were included in the analysis, and the follow-up time ended at December 2016. The inclusion criteria was patients confirmed as IPF after multidisciplinary review in accordance with the American Thoracic Society/European Respiratory Society guideline [11]. We excluded patients who lacked HRCT results, spirometry parameters to diagnose SAD, and results of follow-up (Fig. 1). All patients have signed informed consents, and the present study was approved by the Ethics Committee of Beijing Chao-Yang Hospital.

### Data collection

We collected baseline clinical parameters in hospitalization. Patients were hospitalized for diagnosis or due to acute worsening of pulmonary fibrosis. Demographic information included age, gender and smoking status. Smoking status was categorized as never, current and former smoker. Charlson comorbidity index (CCI) was further calculated according to that suggested by Charlson et al. [12]. Pulmonary function tests were performed after admission to hospital according to standard protocol [13], and baseline spirometry data like percent predicted forced expiratory volume in 1s (FEV1, % predicted), percent predicted forced vital capacity (FVC, % predicted) and percent predicted diffusing capacity of the lungs for carbon monoxide (DLCO, % predicted) was collected, as well as spirometry parameters that used to diagnose SAD. We measured routine blood parameters using fasting venous blood samples taken at the morning during hospitalization, including white blood cell count, neutrophil, lymphocyte, eosinophil, monocyte, red blood cell count, hemoglobin, platelet count and CRP. Blood samples were tested within 4 h after collection. We calculated neutrophil-to-lymphocyte ratio (NLR), platelet-to-lymphocyte ratio (PLR), lymphocyte-to-monocyte ratio (LMR) and monocyte-red blood cell count ratio (MRR) based on routine blood parameters. Survival data were obtained from medical records and telephone interviews, including survival status, cause of death, and time of death.

### Diagnostic criteria of SAD

Three spirometry indicators were used to assess SAD, including maximal mid-expiratory flow (MMEF), forced expiratory flow (FEF) at 50% of vital capacity (FEF 50%), and FEF at 75% of vital capacity (FEF 75%). To be consistent with previous studies [4, 14–17], we defined SAD as at least two of these three indicators were less than 65% of predicted values.

### Statistical analysis

All methods were carried out in accordance with relevant guidelines and regulations. Continuous variables were presented as mean ± sd, and categorical variables were presented as frequency (percentage). We used the Student’s *t* test or Chi-square to compare the differences between patients with and without SAD. We used the Kaplan–Meier to compare survival rate, and the Log-rank tests to compare survival time. Logistic regression analysis was conducted to identify the predictor of SAD. Optimal cut-off points were determined using ROC analysis. Unadjusted and adjusted COX proportional hazard models were used to calculate hazard ratio (HR) and 95% confidence interval (CI) after checking proportional hazards assumption by Weighted Schoenfeld residuals. All statistical tests were two-sided, and performed at the 0.05 significance level. All statistical analyses were conducted by SAS software, version 9.4 (SAS Institute Inc.).

## Results

### Basic information of study subjects

A total of 416 patients with IPF were included in the study, with the mean age being 65.09 years. Among them, 165 (39.66%) patients had SAD, and 117 (37.99%) in 308 patients without other pulmonary comorbidities (including COPD, emphysema and asthma). In 159 patients with normal lung function (FEV1 ≥ 80 predict and FEV1/FVC ≥ 0.7), 15.72% patients had SAD. Results of differences in patient characteristics between IPF patients with SAD and without SAD were shown in Table 1. Smoking status (*p* = 0.019) showed significant difference between IPF patients with SAD and without SAD, IPF patients with SAD had higher percentage of current smoker. There was no significant difference in age and gender.

**Table 1:** Clinical characteristic differences between patients with SAD and without SAD

Characteristics	Without SAD n = 251	With SAD n = 165	*p*
Age (years)	64.89 ± 9.71	65.39 ± 8.83	0.598
Males	210 (83.67)	141 (85.45)	0.623
Smoking			
Non-smoker	81 (32.27)	41 (24.85)	0.019
Current smoker	61 (24.30)	61 (36.97)	
Former smoker	109 (43.43)	63 (38.18)	
Emphysema	55 (21.91)	41 (24.85)	0.487
COPD	7 (2.79)	16 (9.70)	0.003
PTE	0 (0.00)	2 (1.21)	0.080
Pulmonary arterial hypertension	22 (8.76)	15 (9.09)	0.909
Pulmonary infection	47 (18.73)	44 (26.67)	0.055
Respiratory failure	34 (13.55)	28 (16.97)	0.337
Asthma	3 (1.20)	2 (1.21)	0.988

Data are expressed as mean ± sd or count (percentage) where appropriate. *P* was calculated by the Student’s t-test for continuous variables and the Chi-square test and Fisher’s exact test for categorical variables

### Clinical features of IPF patients with or without SAD

**Fig. 2:**
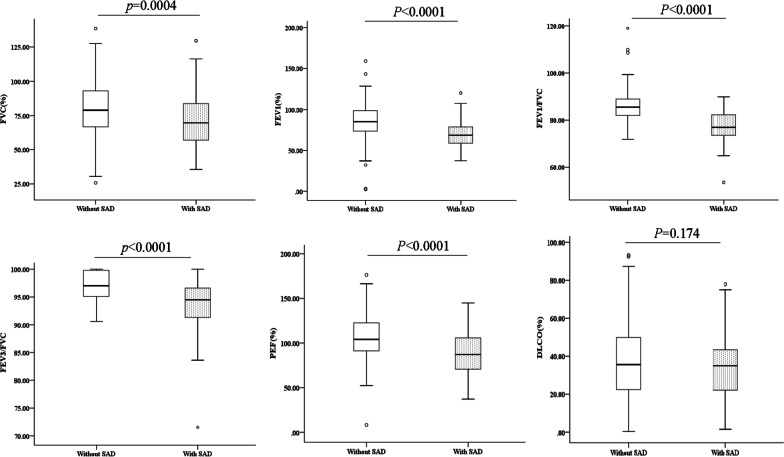
Differences in spirometry parameters between IPF patients with and without SAD

Compared with IPF patients without SAD, IPF patients with SAD had significantly lower FEV1 (% predicted, 69.24 vs. 85.16, *p* < 0.0001), FVC (% predicted, 71.43 vs. 79.47, *p* = 0.0004), FEV1/FVC (77.28 vs. 86.13, *p* < 0.0001), FEV3/FVC (94.00 vs. 97.02, *p* < 0.0001) and PEF (87.91 vs. 106.61, *p* < 0.0001). DL_CO_ did not show significant difference (Fig. 2).

There was significantly higher percentage of COPD in IPF patients with SAD (9.70% vs. 2.79%, *p* = 0.003). IPF patients with SAD had higher percentage of pulmonary infection (26.67% vs. 18.73%, *p* = 0.055) and arrhythmia (6.06% vs. 2.39%, *p* = 0.057), the difference was almost statistically significant. There was no significant difference in the other comorbidities (Table 1, Additional file 1: Table S1).

IPF patients with SAD more frequently presented with moist rales (30.52% vs 19.32%, *p* = 0.014). There was no significant difference in the other symptoms or physical signs.

Significantly higher NLR (3.20 vs. 2.62, *p* = 0.010), higher PLR (133.58 vs. 105.27, *p* < 0.0001) and lower LMR (3.96 vs. 4.44, *p* = 0.043) were found in IPF patients with SAD (Additional file 1: Table S1).

Results of arterial blood gas test were compared between IPF patients with and without SAD. FiO_2_ (5.64 mmHg vs. 3.89 mmHg, *p* = 0.0019) were higher in IPF patients with SAD than those without, whereas PaO_2_ (73.60 vs. 79.30, *p* = 0.0004) were lower (Additional file 1: Table S1).

We compared HRCT characteristics between IPF patient with SAD and without SAD, and no significant difference was found (Additional file 1: Table S1).

### Risk factor analysis for IPF patients with SAD

We did univariate logistic regression analysis of clinical parameters to evaluate the predictors for SAD in patients with IPF (Additional file 1: Figure S1), and found that smoking, FEV1 (% predicted), FVC (% predicted), FEV1/FVC (%), FEV3/FVC (%), PEF, complicated with COPD, moist rales, NLR, PLR, LMR, blood FiO_2_ and PaO_2_ were significantly associated with SAD in patients with IPF (Table 2). These significant characteristics in univariate logistic regression analysis were used to do multivariable analysis. As shown in Table 2, FEV1 (% predicted) and FEV3/FVC (%) were significant predictors for SAD in patients with IPF. Gender was also associated with SAD.

**Table 2:** Univariate and multivariate analysis of predictive factors for SAD

Characteristics	Univariate analysis		Multivariate analysis	
	OR (95% CI)	*p*	OR (95% CI)	*p*
Age	1.01 (0.98–1.03)	0.597	0.99 (0.94–1.05)	0.776
Males	0.87 (0.50–1.51)	0.623	5.94 (1.04–33.97)	0.045
Smoking				
Non-smoker	Reference		Reference	
Current smoker	1.98 (1.18–3.31)	0.010	2.79 (0.62–12.60)	0.183
Former smoker	1.14 (0.70–1.86)	0.594	2.41 (0.61–9.58)	0.210
COPD	3.74 (1.50–9.31)	0.005	3.97 (0.32–49.32)	0.284
Moist rales	1.83 (1.13–2.98)	0.015	2.61 (0.82–8.27)	0.103
FEV1, % predicted	0.95 (0.94–0.97)	< 0.0001	0.89 (0.86–0.93)	< 0.0001
FEV3/FVC, %	0.73 (0.66–0.80)	< 0.0001	0.53 (0.42–0.66)	< 0.0001
NLR	1.12 (1.03–1.23)	0.012	1.07 (0.69–1.67)	0.760
PLR	1.01 (1.00–1.01)	< 0.0001	1.00 (0.99–1.02)	0.563
LMR	0.87 (0.76–1.00)	0.045	1.05 (0.76–1.44)	0.776
PaO2	0.97 (0.96–0.99)	0.001	1.00 (0.97–1.04)	0.849
FiO2	1.04 (1.01–1.06)	0.006	0.97 (0.82–1.14)	0.682

To make the evaluation of these predictors more practical, we have found the optimal cut-off values for spirometry indicators. The optimal cut-off values for FEV1 (% predicted), FVC (% predicted), FEV1/FVC (%), FEV3/FVC (%), PEF were 78%, 66.4%, 93.02% and 93.2%, respectively. Patients were divided into two groups according to the cut-off values. Patients with indicators more than the cut-off values were treated as the high group, whereas, patients with indicators less than or equal to the cut-off values were treated as the low group. Among these spirometry indicators, FEV1/FVC had the highest numerical area under the curve (AUC) (Additional file 1: Figure S2). The results for univariate and multivariate logistic regression analysis of the indicators as categorical variables for SAD were shown in Table 3 and Additional file 1: Table S2. FEV1 (% predicted) and FEV3/FVC (%) as categorical variables were significant predictors for SAD in patients with IPF.

**Table 3:** Multivariable analysis of the risk factors as categorical variables for SAD

Characteristics	Univariate analysis		Multivariate analysis	
	OR (95% CI)	*p*	OR (95% CI)	*p*
FEV1, % predicted				
High group	Reference		Reference	
Low group	5.57 (3.38–9.18)	< 0.0001	30.04 (9.61–93.90)	< 0.0001
FEV3/FVC, %				
High group	Reference		Reference	
Low group	18.94 (8.53–42.07)	< 0.0001	77.76 (15.44–391.63)	< 0.0001

High group was defined as the result more than the cut-off value; Low group was defined as the result less than or equal to the cut-off value. OR for multivariate analysis was adjusted for age, gender, smoking, COPD, Moist rales, NLR, PLR, LMR, PaO2 and FiO2, FEV1 (% predicted), FEV3/FVC (% predicted)

### Prognosis analysis of SAD on survival in patients with IPF

**Fig. 3:**
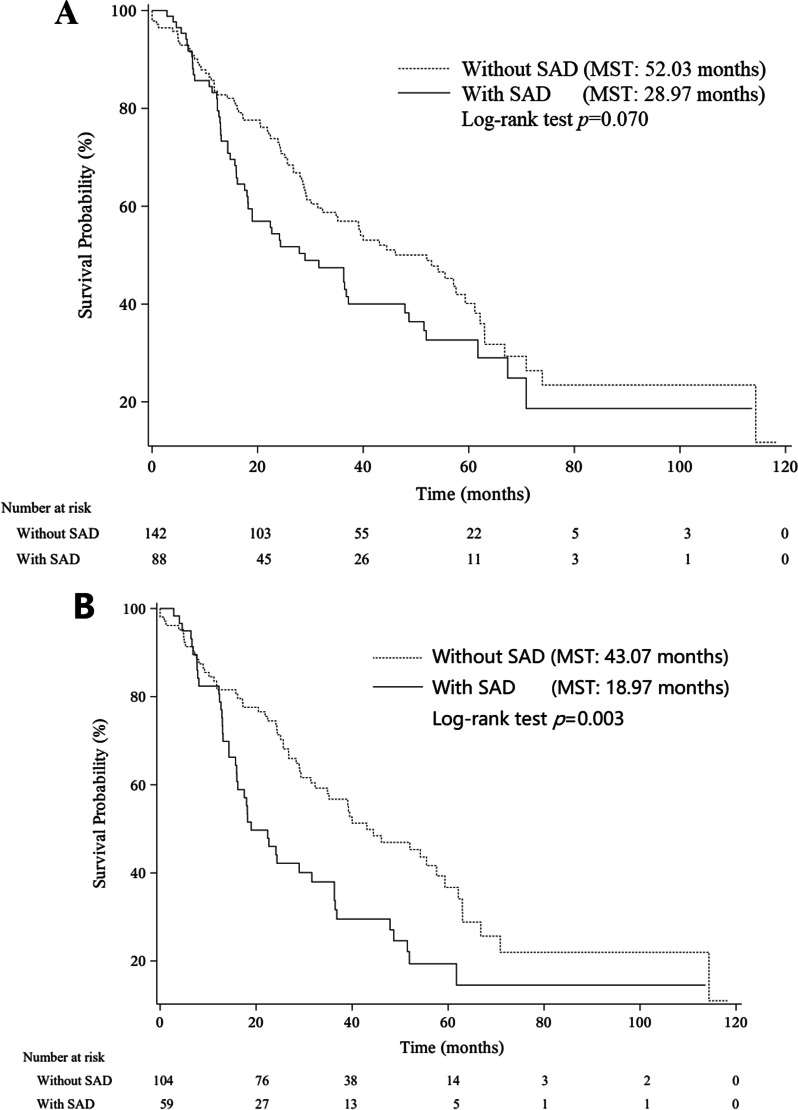
Kaplan–Meier curves of SAD in IPF patients. **A** Kaplan–Meier curve of SAD in overall patients; **B** Kaplan–Meier curve of SAD in patients without other pulmonary comorbidities (COPD, emphysema, and asthma)

After analyzing the predictors for SAD, we analyzed the relationship between SAD and prognosis in patients with IPF. Among all the participants in the study, 227 patients had results of follow-up and were finally included in the survival analysis. The follow-up time ranged from 0.83 months to 118.17 months (median: 28.97 months). At the end of follow-up, a total of 130 deaths occurred with median survival time (MST) being 39.10 months (IQR: 16.13–70.87 months). As shown in Fig. 3, MST for patients with SAD was 28.97 months (IQR: 13.00–67.37 months), and was significantly shorter than that for patients without SAD (MST: 52.03 months, IQR: 22.10–73.93 months, log-rank *p* = 0.038).

First, the assumption of proportional hazards was satisfied tested by Weighted Schoenfeld residuals. Age, gender, smoking status, FEV1/FVC (%), DL_CO_ (%), CCI, drug therapy (hormone, theophylline, N-acetylcysteine, seretide) were treated as confounding factors. After adjusting for these confounding factors, patients with SAD were associated with significantly increased risk of mortality, and the HR (95% CI) was 1.73 (1.02–2.92) as shown in Table 4. We also analyzed the relationship between SAD and prognosis in IPF patients without other pulmonary comorbidities (COPD, emphysema, and asthma), and found that survival of patients with SAD were also poorer than patients without SAD (MST: 18.97 months vs. 43.07 months, log-rank *p* = 0.003, HR 1.93, 95% CI 1.00–3.73) (Fig. 3). SAD was still associated with poorer prognosis in smokers (HR 2.01, 95% CI 1.05–3.84).

**Table 4:** Multivariate risk prediction of SAD for mortality in patients with IPF

Characteristics	HR (95% CI)	*p*
SAD	1.73 (1.02–2.92)	0.040
Age	0.99 (0.96–1.01)	0.295
Males	1.62 (0.83–3.16)	0.158
FEV1/FVC, %	1.02 (0.98–1.06)	0.289
DL_CO_, %	0.99 (0.97–1.00)	0.036
Smoking		
Non-smoker	Reference	
Current smoker	0.87 (0.45–1.68)	0.683
Former smoker	0.79 (0.43–1.47)	0.460
CCI	1.11 (0.89–1.38)	0.352
Hormone	1.46 (0.82–2.62)	0.199
Theophylline	1.03 (0.57–1.87)	0.910
N-acetylcysteine	0.98 (0.61–1.58)	0.933
Seretide	1.01 (0.58–1.75)	0.970

HR (95% CI) was calculated by the COX proportional hazard model

## Discussion

In this present study, we aimed to explore the predictor for SAD, and further investigate the prognosis effect of SAD in Chinese patients with IPF. Via comprehensive analysis, we found that patients with lower FEV1 (% predicted) and FEV3/FVC had increased risk for SAD. IPF patients with SAD were more likely to have poorer prognosis.

In the recent years growing interest has focused on the involvement of small airway in various bronchiolar and interstitial lung diseases [18–21]. Previous studies have found histopathological evidence for SAD in patients with IPF. In 1977, Fulmer and colleagues found that morphologic and physiologic abnormalities of small airway were present in IPF, and linked pathology in the small airways to abnormal lung physiology in patients with IPF for the first time [7]. Since then, IPF is a disease of alveoli as well as small airways. Via comprehensive analysis of lung biopsies from patients with IPF using a cascade of clinical multi-detector CT (MDCT) scan, Verleden et al. [8] found that thickening of small airway walls and distortion of small airway lumens could increase the visibility of small airways on MDCT scans. Comparing with normal lung anatomy, the number of terminal bronchioles reduced 60% in regions of minimal fibrosis in IPF lungs, however, this number did not show further decline in regions of established fibrosis. Our previous study also revealed that patients with IPF have abnormal pathologic, physiological and imaging changes of small airways [10]. In our current study, almost 40% of patient with IPF have been diagnosed as SAD. Even 38% patients had SAD in patients without other pulmonary comorbidities (including COPD, emphysema and asthma). 18% of patients whose lung function was normal (FEV1 ≥ 80 predict and FEV1/FVC ≥ 0.7) had SAD, indicating that SAD might also be a precursor of the change of large airway in patients with IPF.

Xiao et al. [4] explored the risk factors for SAD in a large Chinese population chose by a multistage stratified sampling method, and found that cigarette smoking was a major modifiable risk factor, along with PM2·5 exposure and increase of BMI by 5 kg/m^2^. However, risk factor for SAD in patients with IPF was not clear. In our current study, patients with SAD showed higher percentage of current smoker. We believe that stronger tobacco control is needed to improve lung health and slower the progression of disease in patient with IPF. Multivariate analysis found that IPF patients with lower FEV1 (% predicted) and FEV3/FVC were more likely to have SAD.

The prognosis of patients with IPF was heterogeneous, and the staging of disease was difficult. In our current study, complicated with SAD could increase the risk of mortality in patients with IPF, as well as in patients without other pulmonary comorbidities. Hu et al. [22] used impulse oscillometry to detect SAD and found that FEV1, FEF 25%-75%, and CAT score improved significantly after bronchodilator treatment in IPF patients with SAD, while bronchodilator efficacy was not observed in those without, indicating that functional parameters of small airways could guide bronchodilator use in IPF. Previously, FEV1/FVC < 0.7 was a criterion for the use of inhaled bronchodilators in IPF, however, most of patients with IPF have FEV1/FVC > 0.8 as shown in previous studies. In our current study, almost 70% of patients have FEV1/FVC > 0.8, and 17% of them had SAD. SAD may be useful for guiding bronchodilator therapy and grading the disease.

There were many methods used to diagnose SAD, including spirometry, forced oscillation technique, nitrogen washout test, peripheral wedged catheters, PET, and MRI [23]. So far there was no gold standard. In our current study, we used lung function parameters to detect SAD, as it was the most widely used and non-invasive method. Several spirometry parameters were used for defining SAD, such as FEF25-75%, MMEF, FEV3/FVC, and so on [24–26]. Among them, FEF 25–75% is dependent on the FVC and frequently normal when the FEV1/FVC is more than 75%, therefore its reproducibility and sensitivity are limit [23]. The difference in diagnostic criteria also lead to difference in SAD incidence. There was a national cross-sectional study conducted in China to explore the prevalence and risk factors of SAD. Three parameters (MMEF, FEF 50%, and FEF 75%) were used to define SAD in this study and other previous studies, including those conducted in the Chinese population [4, 14–17, 27–29]. In addition, SAD was defined by this criterion in Chinese guideline [30]. To be compatible with previous studies, especially in the Chinese population, we used MMEF, FEF 50%, and FEF 75% to diagnose SAD. SAD is early manifestations of airway obstruction. The curve of maximum mid-expiratory flow is in the non-force dependent part of FVC, that is, the expiratory flow is fixed despite increasing the force when the force degree reaches a certain limit. The flow of low lung volume is affected by small airway diameter, and the decrease of flow reflects the obstruction of small airway. Therefore, at the early stage of SAD, MMEF, FEF 50%, and FEF 75% decreased significantly, while there could be rarely symptom or sign, and the value of FVC, FEV1, and FEV1/FVC could be in normal range [30].

There were several potential limitations in the current study. Firstly, our diagnosis of SAD was based on spirometry, which is more practical. Therefore, our findings could be applied only to spirometry-defined SAD. Secondly, this was a single-center study, and multi-center study will be conducted in the future to demonstrate the effect of SAD in IPF.

## Conclusions

In conclusion, we found that spirometry-defined SAD was like 40% in patients with IPF. Patients with lower FEV1 (% predicted) and FEV3/FVC were more likely to have SAD. IPF patients with SAD showed poorer prognosis. SAD will be helpful in managing and grading the patients with IPF in the future.

## Supplementary Information

**Additional file 1: Table S1.** Clinical characteristic differences between patients with SAD and without SAD. **Table S2.** Univariate logistic regression analysis of the risk factors as categorical variables for SAD. **Figure S1.** Forest plot of univariate analysis of the risk factor as continuous variables for SAD. **Figure S2.** ROC curves of spirometry parameters in predicting SAD. A, FEV1; B, FVC; C, FEV1/FVC; D, FEV3/FVC; E, PEF.

## Data Availability

The datasets generated and/or analysed during the current study are not publicly available but are available from the corresponding author on reasonable request.

## Abbreviations

SAD: Small airway dysfunction
HR: Hazard ratio
CI: Confidence interval
IPF: Idiopathic pulmonary fibrosis
FEV1: Forced expiratory volume in 1s
FVC: Forced vital capacity
DL_CO_: Diffusing capacity of the lungs for carbon monoxide
NLR: Neutrophil-to-lymphocyte
PLR: Platelet-to-lymphocyte ratio
LMR: Lymphocyte-to-monocyte ratio
MRR: Monocyte-red blood cell count ratio
