# Supplementary material for: Small airway dysfunction in Chinese patients with idiopathic pulmonary fibrosis
Source: BMC Pulm Med. 2022 Aug 2;22:297. doi: 10.1186/s12890-022-02089-6 (PMC9347131; doi:10.1186/s12890-022-02089-6)

Table S1 Clinical characteristic differences between patients with SAD and without SAD

| Characteristics | Without SAD  n=251 | With SAD  n=165 | *p* |
| --- | --- | --- | --- |
| Comorbidity |  |  |  |
| Diabetes mellitus | 40 (15.94) | 27 (16.36) | 0.908 |
| Hypertension | 60 (23.90) | 48 (29.09) | 0.238 |
| Coronary heart disease | 44 (17.53) | 21 (12.73) | 0.187 |
| Arrhythmia | 6 (2.39) | 10 (6.06) | 0.057 |
| Connective tissue disease | 5 (1.99) | 1 (0.61) | 0.219 |
| Pulmonary heart disease | 10 (3.98) | 9 (5.45) | 0.482 |
| Gastroesophageal reflux | 26 (10.36) | 9 (5.45) | 0.078 |
| Tuberculosis | 4 (1.59) | 6 (3.64) | 0.190 |
| Cancer | 7 (2.79) | 6 (3.64) | 0.627 |
| Symptom |  |  |  |
| Dyspnea | 154 (73.33) | 114 (73.08) | 0.956 |
| Cough | 198 (93.40) | 144 (91.72) | 0.541 |
| Expectoration | 166 (77.93) | 127 (81.94) | 0.347 |
| Chest pain | 11 (5.29) | 15 (9.80) | 0.101 |
| Chest distress | 38 (18.10) | 34 (22.22) | 0.330 |
| Physical sign |  |  |  |
| Erythra | 6 (2.83) | 5 (3.18) | 0.844 |
| Cyanosis | 48 (22.97) | 46 (30.07) | 0.128 |
| Moist rales | 40 (19.32) | 47 (30.52) | 0.014 |
| Velcro | 152 (81.72) | 89 (74.79) | 0.147 |
| Acropachia | 86 (41.95) | 49 (32.89) | 0.083 |
| Redness and swelling of joints | 0 | 0 | / |
| Dysarthrose | 3 (1.41) | 0 (0.00) | 0.067 |
| Lower Limb Edema | 21 (9.86) | 11 (7.10) | 0.353 |
| HRCT |  |  |  |
| Pleural thickening | 100 (75.76) | 56 (74.67) | 0.861 |
| Node | 28 (17.83) | 13 (14.94) | 0.563 |
| Grid shadow | 114 (86.36) | 59 (78.67) | 0.151 |
| Honeycomb | 55 (33.95) | 32 (35.56) | 0.797 |
| Ground glass | 99 (60.37) | 61 (67.78) | 0.242 |
| Consolidation shadows | 5 (3.09) | 5 (5.62) | 0.337 |
| Interlobular septa thickened | 85 (52.15) | 52 (59.09) | 0.292 |
| Widening of pulmonary artery | 9 (5.66) | 4 (4.55) | 0.704 |
| Mediastinal adenopathy | 88 (53.33) | 53 (59.55) | 0.342 |
| Pleural effusion | 53 (32.12) | 30 (33.71) | 0.797 |
| Lymph node calcification | 32 (24.24) | 19 (25.33) | 0.861 |
| Bullae | 62 (37.80) | 35 (39.33) | 0.812 |
| Aortosclerosis | 94 (71.21) | 48 (64.00) | 0.283 |
| Blood routine |  |  |  |
| NLR | 2.62 ± 2.05 | 3.20 ± 2.39 | 0.010 |
| PLR | 105.27 ± 47.60 | 133.58 ± 81.18 | <0.0001 |
| LMR | 4.44 ± 1.89 | 3.96 ± 1.88 | 0.043 |
| CRP | 4.55 ± 18.57 | 5.00 ± 17.58 | 0.828 |
| AGR | 1.05 ± 0.25 | 1.07 ± 0.24 | 0.566 |
| LDH | 202.92 ± 52.37 | 198.05 ± 54.59 | 0.385 |
| Blood gas |  |  |  |
| FiO_2_ | 2.89 ± 7.54 | 5.64 ± 10.00 | 0.005 |
| PaCO_2_ | 39.33 ± 9.34 | 39.10 ± 5.50 | 0.789 |
| PaO_2_ | 79.30 ± 15.62 | 73.60 ± 13.46 | 0.0004 |
| SaO_2_ | 94.35 ± 7.51 | 93.87 ± 3.84 | 0.476 |
| SBE | 1.10 ± 2.03 | 1.31 ± 2.30 | 0.418 |
| Oxygenation index | 369.31 ± 84.58 | 349.54 ± 75.77 | 0.053 |

Data are expressed as mean ± sd or count (percentage) where appropriate. *P* was calculated by the t-test for continuous variables and the Chi-square test and Fisher’s exact test for categorical variables.

Table S2 Univariate logistic regression analysis of the risk factors as categorical variables for SAD

| Characteristics | OR (95% CI) | *p* |
| --- | --- | --- |
| FEV1, % predicted |  |  |
| High group | reference |  |
| Low group | 5.57 (3.38-9.18) | <.0001 |
| FVC, % predicted |  |  |
| High group | reference |  |
| Low group | 2.49 (1.55-4.01) | 0.0002 |
| FEV1/FVC |  |  |
| High group | reference |  |
| Low group | 13.90 (7.98-24.21) | <.0001 |
| FEV3/FVC |  |  |
| High group | reference |  |
| Low group | 18.94 (8.53-42.07) | <.0001 |
| PEF |  |  |
| High group | reference |  |
| Low group | 4.01 (2.50-6.43) | <.0001 |

Note: The cut-off values for continuous variables were: 78 for FEV1; 66.4 for FVC; 93.02 for FEV1/FVC; 93.02 for FEV3/FVC; 93.2 for PEF.

Figures

Figure S1. Forest plot of univariate analysis of the risk factor as continuous variables for SAD.


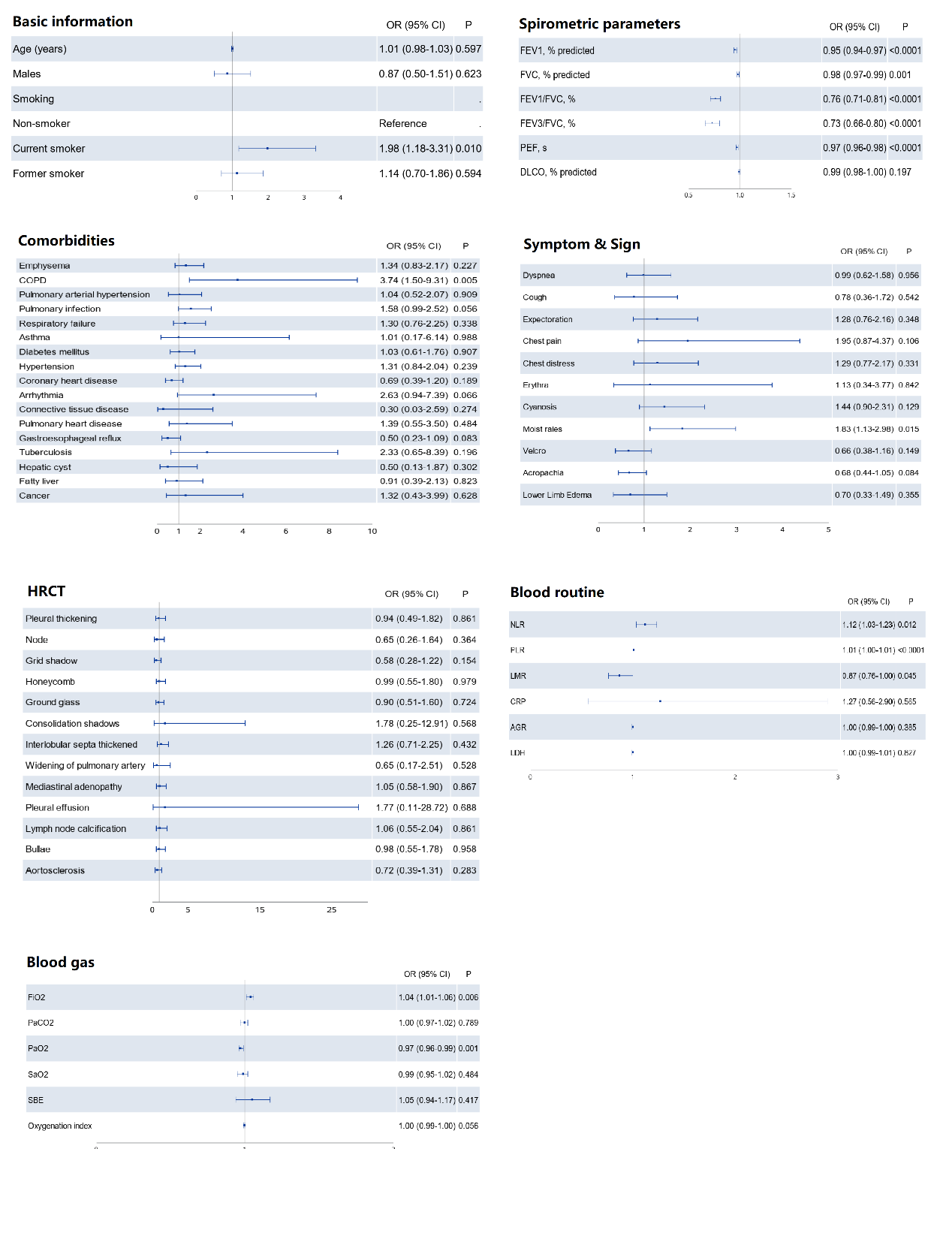


Figure S2. ROC curves of spirometry parameters in predicting SAD. A, FEV1; B, FVC; C, FEV1/FVC; D, FEV3/FVC; E, PEF.


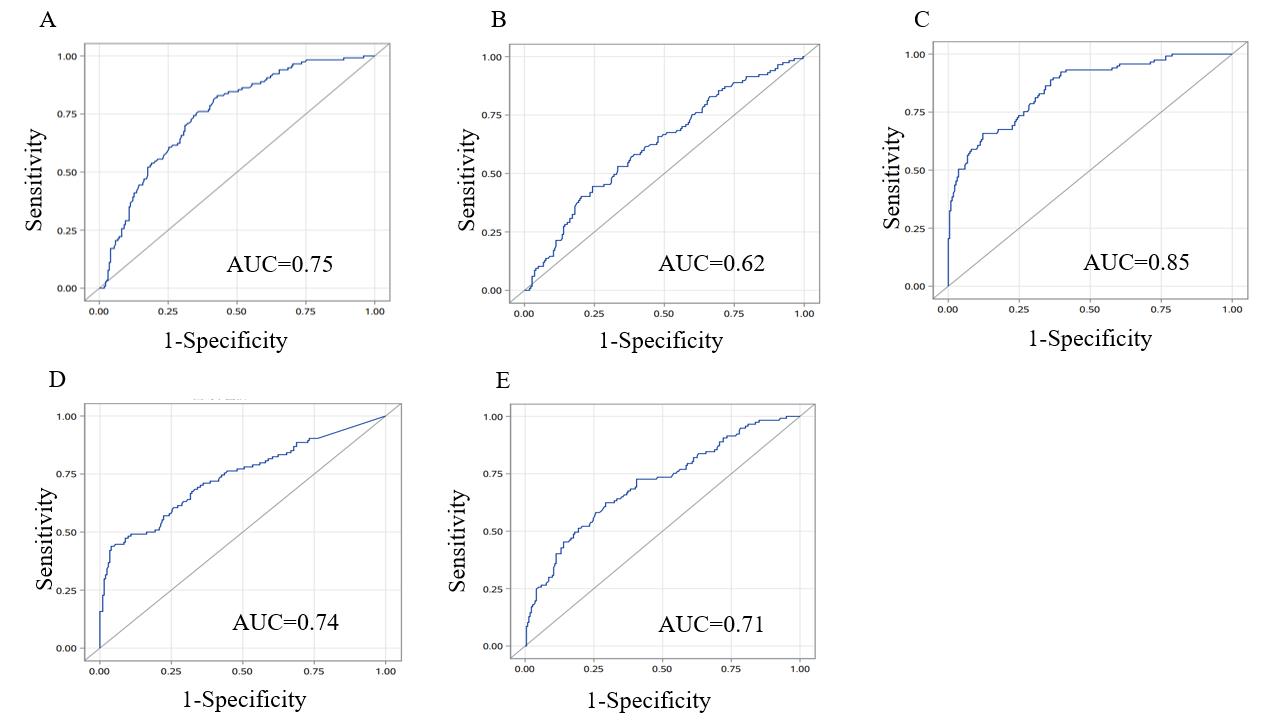

Supplement: Supplementary file 1 — Additional file 1: Table S1. Clinical characteristic differences between patients with SAD and without SAD. Table S2. Univariate logistic regression analysis of the risk factors as categorical variables for SAD. Figure S1. Forest plot of univariate analysis of the risk factor as continuous variables for SAD. Figure S2. ROC curves of spirometry parameters in predicting SAD. A, FEV1; B, FVC; C, FEV1/FVC; D, FEV3/FVC; E, PEF. [file 12890_2022_2089_MOESM1_ESM.docx]
